# Exosome-Mediated Paracrine Signaling Unveils miR-1246 as a Driver of Aggressiveness in Fusion-Negative Rhabdomyosarcoma

**DOI:** 10.3390/cancers16091652

**Published:** 2024-04-25

**Authors:** Farah Ramadan, Raya Saab, Farah Ghamloush, Rita Khoueiry, Zdenko Herceg, Ludovic Gomez, Bassam Badran, Philippe Clezardin, Nader Hussein, Pascale A. Cohen, Sandra E. Ghayad

**Affiliations:** 1 Université Lyon 1, Lyon, France; farah.ramadan@univ-lyon1.fr (F.R.); philippe.clezardin@inserm.fr (P.C.); 2 INSERM, Research Unit UMR_S1033, LyOS, Faculty of Medicine Lyon-Est, 69372 Lyon, France; 3 Department of Biology, Faculty of Science II, Lebanese University, Beirut 6573, Lebanon; 4 Laboratory of Cancer Biology and Molecular Immunology, Department of Chemistry and Biochemistry, Faculty of Science I, Lebanese University, Hadath 1103, Lebanon; bassam.badran@ul.edu.lb (B.B.); nader.hussein@lyon.unicancer.fr (N.H.); 5 Department of Pediatrics & Adolescent Medicine, American University of Beirut Medical Center, Beirut 1107, Lebanon; rsaab@stanford.edu (R.S.); fg07@aub.edu.lb (F.G.); 6 Department of Anatomy, Cell Biology and Physiology, Faculty of Medicine, American University of Beirut, Beirut 1107, Lebanon; 7 Department of Pediatrics, Stanford University School of Medicine, Palo Alto, CA 94304, USA; 8 Epigenomics and Mechanisms Branch, International Agency for Research on Cancer, World Health Organization, 69366 Cedex 07 Lyon, France; khoueiryr@iarc.who.int (R.K.); hercegz@iarc.who.int (Z.H.); 9 Laboratoire CarMeN—IRIS Team, INSERM, INRA, Université Claude Bernard Lyon-1, INSA-Lyon, Univ-Lyon, 69500 Bron, France; ludovic.gomez@univ-lyon1.fr; 10 Centre de Recherche en Cancérologie de Lyon, INSERM U1052, CNRS UMR 5286, Centre Léon Bérard, Université Lyon 1, 69008 Lyon, France; 11 C2VN, INSERM 1263, INRAE 1260, Aix-Marseille University, 13005 Marseille, France; 12 Department of Pharmaceutical Biology, Faculty of Pharmacy, Aix-Marseille University, 27 Boulevard Jean Moulin, 13005 Marseille, France

**Keywords:** Rhabdomyosarcoma, fusion-negative RMS, exosomes, miR-1246, paracrine signaling, aggressiveness

## Abstract

**Simple Summary:**

Rhabdomyosarcoma (RMS) is a rare cancer that occurs in children and adolescents. The presence of metastasis is associated with poor outcomes and highlights the need for new non-invasive therapies. Exosomes are small extracellular vesicles that have an important impact on disease biology by altering the behavior of recipient cells. RMS-derived exosomes are enriched in miRNAs which can regulate tumorigenesis and metastasis. This study newly identifies miR-1246 as a key miRNA enriched in the cargo of exosomes derived from RMS cells. MiR-1246 has the potential to be delivered to recipient fibroblasts, effectively altering their phenotype and contributing to their aggressiveness. In a pioneer clinical study, miR-1246 in the serum exosomes of RMS patients was found to be enriched. Our results pave the way for the use of exosomal miR-1246 as a key mediator of cell aggressiveness in RMS, thus representing an attractive future therapeutic target.

**Abstract:**

Rhabdomyosarcoma is a pediatric cancer associated with aggressiveness and a tendency to develop metastases. Fusion-negative rhabdomyosarcoma (FN-RMS) is the most commonly occurring subtype of RMS, where metastatic disease can hinder treatment success and decrease survival rates. RMS-derived exosomes were previously demonstrated to be enriched with miRNAs, including miR-1246, possibly contributing to disease aggressiveness. We aimed to decipher the functional impact of exosomal miR-1246 on recipient cells and its role in promoting aggressiveness. Treatment of normal fibroblasts with FN-RMS-derived exosomes resulted in a significant uptake of miR-1246 paired with an increase in cell proliferation, migration, and invasion. In turn, delivery of miR-1246-mimic lipoplexes promoted fibroblast proliferation, migration, and invasion in a similar manner. Conversely, when silencing miR-1246 in FN-RMS cells, the resulting derived exosomes demonstrated reversed effects on recipient cells’ phenotype. Delivery of exosomal miR-1246 targets *GSK3β* and promotes β-catenin nuclear accumulation, suggesting a deregulation of the Wnt pathway, known to be important in tumor progression. Finally, a pilot clinical study highlighted, for the first time, the presence of high exosomal miR-1246 levels in RMS patients’ sera. Altogether, our results demonstrate that exosomal miR-1246 has the potential to alter the tumor microenvironment of FN-RMS cells, suggesting its potential role in promoting oncogenesis.

## 1. Introduction

RMS is a malignant pediatric soft tissue sarcoma, originating from primitive mesenchymal cells with a tendency for myogenic differentiation, that can occur sporadically and at different sites in the body [1]. Current classification of RMS is primarily based on fusion status, that is the presence or absence of a fusion oncoprotein, most commonly PAX3-FOXO1 [2,3]. Fusion-negative RMS (FN-RMS) comprises the majority of RMS cases (80%), of which embryonal RMS (ERMS) is the most commonly occurring histologic subtype, comprising 60% of RMS cases [1]. While associated with a favorable prognosis and overall survival rates that can reach 90% with multimodal treatment strategies, high-risk groups and patients with metastatic or recurrent disease still experience adverse outcomes and overall survival rates as low as 25% [4,5]. For example, the 5-year event-free survival after relapse of Stage 4 ERMS patients is 12% compared with 50% for patients with Stage 1–Group I ERMS [6]. Thus, it is of utmost interest to delineate the molecular events contributing to FN-RMS disease aggressiveness.

Exosome-mediated paracrine signaling, whereby cancer-derived exosomes deliver nucleic acids and proteins to recipient cells including stromal cells in the tumor microenvironment and cells in distant areas, can promote disease aggressiveness and prime distant sites for metastasis [7,8]. These small extracellular nanovesicles have emerged as important contributors to cancer growth, invasion, and metastasis [9,10,11]. In RMS, exosomes were shown to promote recipient fibroblasts’ proliferation, migration, and invasive capacities, indicating presence of protein and nucleic acids within the exosomal cargo that could mediate the observed paracrine-signaling events [12]. Generally, exosomes carry a plethora of miRNA and have been investigated for their correlation with cancer development, stages of disease, or presence of metastasis [13,14]. However, studies investigating the role of FN-RMS-derived exosomes are still very few [10,12,15,16,17], and little is known concerning the exosomal cargo that contributes to FN-RMS progression. We showed that in RMS, exosomes carry mostly small-sized RNA cargo, allowing the focus on enriched miRNA cargo to find important players in RMS progression [12].

In that previous work, microarray analysis revealed the common upregulation of miR-1246 in exosomes derived from RMS cells of different fusion status, including three different FN-RMS cell lines [12]. While cellular miR-1246 has exhibited both tumor-suppressive and onco-promoting roles [18], it predominantly acts as an oncomiR in different types of cancer, including pancreatic [19], colorectal [20], and lung cancer [21,22]. It has been suggested to possess superior biomarker value compared with conventional biomarkers for these cancers, based on its specificity and sensitivity values [18]. Exosomal miR-1246 was shown to enhance the migration and invasive action of breast cancer cells [23]. Nevertheless, studies on the exosomal counterpart of this miRNA are still very limited, and its clinical relevance in FN-RMS has not been explored yet.

In this study, we investigated the functional impact of FN-RMS-derived exosomal miR-1246 on recipient fibroblasts, with the aim to decipher its role in altering the tumor stromal microenvironment in a paracrine-signaling manner.

## 2. Materials and Methods

### 2.1. Cell lines and Cell Culture

The human fusion-negative ERMS JR1 cell line was generously donated by Dr. Peter Houghton (Columbus, OH, USA) and the human fusion-negative ERMS RD cell line was purchased from ATCC (Manassas, VA, USA). BJ (human foreskin fibroblast), IMR90 (human fetal lung fibroblast), WI-38 (human fetal lung fibroblast), and HEK293T (human embryonic kidney) cell lines were purchased from ATCC. JR1 and BJ cells were cultured in Dulbecco’s Modified Eagle’s Medium AQ (DMEM AQ, Sigma-Aldrich, Dorset, UK), while IMR90 and WI-38 cells were culture in Eagle’s Minimum Essential Medium (EMEM, Sigma-Aldrich). All media contained 10% fetal bovine serum (FBS, Sigma-Aldrich) and 1% penicillin–streptomycin antibiotics (Sigma-Aldrich). Cells were incubated under standard conditions and passaged twice weekly using trypsin–EDTA (Sigma-Aldrich).

For conditioned media collection, cells were grown in medium supplemented with exosome-free (EF) medium which was prepared via ultracentrifugation of DMEM supplemented with 40% FBS at 100,000 g for 18 h at 4 °C, discarding the pellet, and filtering the supernatant through a 0.22 μm filter (Millipore, Darmstadt, Germany). It was then diluted so that the final EF media contained 10% FBS for use in subsequent assays.

### 2.2. MiR-1246 Functional Inhibition

Viral particles were packaged using a pPACKH1 HIV Lentivector Packaging Kit (SBI, Mountain View, CA, USA) according to the manufacturer’s instructions. Briefly, HEK293T cells were transfected with either MiRZip-1246 (catalog no. CS940MZ-1; SBI) or MiRzip-scr (MZIP000-PA-1 pGreen-Puro Scramble Hairpin Control; SBI). Viral supernatants were collected at two different timepoints: 48 h and 72 h, as described previously [15]. Target cells were then transduced in suspension at 32 °C, 1250× *g* for 1 h with 8 μg/mL polybrene (hexadimethrine bromide; Sigma) [15]. GFP-positive cells were detected under a microscope, followed by selection with puromycin (Cf = 0.5 ug/mL).

### 2.3. Exosome Isolation

When cells reached 70% confluence, JR1 and RD cells were cultured in EF medium for 72 h, as previously described [12]. Briefly, the culture medium was collected and subjected to sequential centrifugation steps (300× *g* for 10 min, 2000× *g* for 20 min, 10,000× *g* for 30 min). Total exosome isolation reagent from cell culture media (Invitrogen, Life technologies, Paisley, UK) was added to the resulting supernatant and incubated overnight at 4 °C. The following day, we performed ultracentrifugation at 100,000× *g* for 70 min at 4 °C. The pellet was then collected, and either treated or not with RNase A (Invitrogen) and Proteinase K (Ambion, Life technologies, Paisley, UK) according to the manufacturer’s instructions, and then washed with 25 mL PBS (phosphate-buffered saline), and centrifuged at 100,000× *g* for 70 min. The final pellet (containing the exosome fraction) was resuspended in either PBS for proliferation, migration, and invasion assays or in RIPA 1X lysis buffer or QIAzol lysis reagent (Qiagen, Hilden, Germany) for protein and RNA extractions, respectively. All treatments with exosomes were performed by adding the corresponding exosomes at 10× concentration, where 1× represents exosomes isolated from an equivalent cell count to those treated, and 10× denotes ten times that quantity [12]. The size and the concentration of the isolated exosomes were assessed using dynamic light scattering (DLS) with a Zetasizer Nano ZS instrument (Malvern Panalytical, Malvern, UK). To ensure reproducibility and standardization, fixed parameter values were employed. Measurements were performed with three acquisitions, each comprising 10 readings.

### 2.4. DOTAP-Mimic Lipoplex Preparation

To deliver miR-1246 mimics or the control (Genepharma, Shanghai, China) to recipient cells via liposomes, N-[1-(2,3-Dioleoyloxy)propyl]-N,N,N-trimethylammonium methyl-sulfate (DOTAP) cationic liposomal transfection reagent (catalog no. 11202375001, Roche Diagnostics, Sigma-Aldrich) was used. DOTAP-miRNA mimic lipoplexes were formed according to the manufacturer’s protocols. Briefly, miR-1246 mimics or negative control mimics were complexed at a ratio of 6 μg DOTAP per μg of mimics in 30 μL of N-2-hydroxyethylpiperazine-N-ethanesulfonic acid-buffered saline (HBS; 20 mM HEPES pH 7.4, 150 mM NaCl) and incubated at room temperature for 15 min. Cells at 60% confluency were incubated with the above lipoplexes at a final concentration of miRNA Cf = 25 nM in culture medium. After 6 h, cells were collected for quantification of miR-1246 or kept for longer for functional analysis.

### 2.5. RNA Extraction and Reverse Transcription Real-Time Polymerase Chain Reaction (RTq-PCR)

Cells and exosomes were lysed using QIAzol Lysis reagent (Qiagen) and total RNA was extracted using phenol–chloroform, as previously described [15]. Reverse transcription was performed using the TaqMan™ microRNA Reverse Transcription Kit (Applied Biosystems, Life Technologies, Paisley, UK) followed by real-time PCR using a customized TaqMan™ small RNA assay for hsa-miR-1246 and for internal control RNU6 (cat no. 4398987; Applied Biosystems) on a CFX96 real-time PCR detection system (Bio-Rad) as per the following: 10 min at 95 °C, 40 cycles of 95 °C for 15 s, and 60 °C for 1 min. For analysis of the results and comparison of relative miRNA expression levels, we used the 2^−ΔΔCt^ method.

For detection of mRNA expression levels of miR-1246 targets, total RNA was reverse transcribed using iScript cDNA Synthesis Kit (Bio-Rad, Hercules, CA, USA) followed by RT-qPCR using SsoAdvanced Universal SYBR Green Supermix (Bio-Rad) for 40 cycles using the following primers: *AXIN2* primer pair: sense, 5′-TGACGGACAGCAGTGTAGATG-3′; antisense, 5′-TTCTCGGGAAATGAGGTAGAG-3′; *GSK3β* primer pair: sense, ACAACAGTGGTGGCAACTCC; antisense, 5′-TTCTTGATGGCGACCAGTTCT-3′; *JARID2* primer pair: sense, 5′-GACACCAAACCCAATCACCAC-3′; antisense, 5′-GTTCAACCTGCCACTGACTT-3′.; *CCNG2* primer pair: sense, 5′-GCTGAAAGCTTGCAACTGCCGAC-3′; antisense, 5′-GGTATCGTTGGCAGCTCAGGAAC-3′; *KMT2D* primer pair: sense, 5′-CTCTGGATGGGATTGATGCT-3′; antisense, 5′-CGTGGCTCTTCCTGTTCTTC-3′. *L32* was used as an internal control with the following primer pair: sense, 5′-CAAGGAGCTGGAAGTGCTGC-3′; antisense: 5′-CAGCTCTTTCCACGATGGC-3′.

### 2.6. Cell Proliferation

For the cell proliferation assay, human IMR90, WI-38, or BJ fibroblasts were seeded onto a 24-well plate at a density of 50,000 cells per well and incubated at 37 °C for 4 h. Subsequently, the medium was removed, and either exosomes (10× concentration) or DOTAP mimics (at a final concentration of 25 nM) were added in 1 mL EF medium and incubated for 72 h. Following incubation, the cells were washed with PBS and harvested for cell counting. Cells were stained using trypan blue and counted manually under the microscope.

### 2.7. Cell Migration and Cell Invasion

BD Falcon™ Cell Culture Inserts with an 8 μm pore size (BD Biosciences, Bedford, MA, USA) were utilized for the Transwell migration assay. These inserts were coated with 10% growth factor-reduced matrigel (Corning, Bedford, MA, USA) at a final concentration of 0.3 mg/mL for the invasion assay. In a 24-well plate, IMR90 or BJ fibroblasts were seeded onto the top chamber (30,000 or 50,000 cells per insert) in 300 μL EF medium. In the bottom chamber, we added 500 μL of serum-free medium. Exosomes (10× concentration), DOTAP control mimics, DOTAP miR-1246 mimics, free control mimics, or free miR-1246 mimics (at a final concentration of 25 nM), were added 4 h later onto the top chamber. For the control, cells were left untreated in EF medium. Then, 24 h later, cells were fixed for migration assays and, 48 h later, for invasion assays, stained with hematoxylin and eosin (H&E), mounted, and photographed [12]. Counting of migrated or invaded cells was performed using ImageJ^®^ software (ImageJ 1.54d).

### 2.8. Protein extraction and Western Blotting

Proteins were extracted from exosomes using RIPA 1X lysis buffer. The mixture was kept on ice for 15 min, sonicated for 10 min, and then centrifuged for 15 min at 15,000× *g* at 4 °C. The supernatant containing the proteins was then collected. Western blotting for exosomal markers was performed using equal amounts of proteins loaded onto 4–15% Mini-PROTEAN TGX Stain-Free precast gels (Bio-Rad). An equal volume of loading buffer (4× Laemmli loading buffer diluted in RIPA 1X) was added to each sample, while β-mercaptoethanol was excluded when detecting tetraspanin levels (non-reducing conditions). Protein migration and transfer to the membrane were performed as previously described [10]. The membrane was blocked to prevent non-specific binding using Azure Fluorescent Blot Blocking Buffer (Azure Biosystems, Dublin, CA, USA). The membrane was incubated with specific primary antibodies: anti-CD63 (cat no. 10628D; Invitrogen), anti-CD81 (cat no. 10630D; Invitrogen), anti-Calnexin (cat no. sc-11397, Santa Cruz Biotechnology, Heidelberg, Germany), and anti-Syntenin-1 (cat no. 22399-1-AP, Proteintech, Munich, Germany) diluted in 3% BSA-TBS1X-0.001% Tween overnight at 4 °C. Secondary antibodies used were goat-anti-mouse IgG IR700 (Azure Biosystems) diluted 1 in 10,000 in the Azure Fluorescent Blot Blocking Buffer for 1 h at room temperature. Band visualization was performed using the Azure Biosystems Western Blot Imaging System 500.

### 2.9. Immunofluorescence

Cells were cultured on coverslips in 12-well plates and treated with 10× exosomes in 10% EF media or with DOTAP-negative control mimics or DOTAP-miR-1246 mimics at a final concentration of 25 nM in 500 µL serum-free media. As a positive control for active Wnt signaling, cells were treated with 3µM GSK3β inhibitor CHIR99021 (Sigma-Aldrich) or vehicle-control DMSO. After 48 h, cells were washed with PBS and fixed with 4% paraformaldehyde (Diapath S.p.A., Martinengo BG, Italy) for 15 min. Cells were then washed and permeabilized with 0.2% Triton X-100 (Sigma-Aldrich). Blocking was performed with 2% BSA for 1 h at room temperature. Cells were washed twice with PBS and each coverslip was incubated with primary antibody against total β-catenin (cat no. 8480, Cell Signaling Technology, Danvers, MA, USA), diluted 1:50 in PBS overnight at 4 °C. The next day, the cells were washed with PBS-0.01%Tween and incubated with Alexa Fluor 488 donkey anti-rabbit IgG (H + L) secondary antibody (Invitrogen) diluted 1:1000 in PBS for 1 h at room temperature. Cells were then washed with PBS-0.01% Tween and then counterstained and fixed on slides with DAPI-Fluoromount-G^®^ (SouthernBiotech, Birmingham, AL, USA). Slides were observed at 40× magnification and images were captured using a Zeiss 880 confocal microscope. Pictures were then analyzed using ImageJ^®^ software (ImageJ 1.54f). Corrected total cell fluorescence, in arbitrary units, was calculated as previously described [24].

### 2.10. Pilot Clinical Study

RMS patient sera (*n* = 26) were obtained from the Children’s Oncology Group (COG, Pittsburgh, PA, USA), collected at diagnosis. Annotated clinical features included 17 males and 9 females. Age range was 0.8–29.9 years, with a median of 9.2 years. Sixteen samples were from patients with advanced/unresectable local tumors, and 5 were from patients with metastatic disease. The molecular subtype was fusion-negative in 10 cases and fusion-positive in 16 cases. Control serum samples (*n* = 16) were obtained from the Tumor Tissue Biorepository at the American University of Beirut Medical Center (AUBMC), overseen by the institutional review board (IRB) for research purposes. Control sera were collected from age-matching pediatric patients with a non-cancer diagnosis, or with a previous diagnosis of cancer other than RMS but who were off treatment and in complete remission at the time of serum collection. Briefly, serum samples (500 μL) were diluted in PBS 1X, and exosomes were isolated through sequential centrifugations and incubation with Exoquick (EXOQ5A-1; SBI) overnight at 4 °C. Exosomes were then pelleted by ultracentrifugation for 2 h at 110,000× *g* and then washed with PBS 1X for another 70 min at the same speed. All centrifugations were carried out at 4 °C. The exosomes were lysed using QIAzol Lysis reagent (Qiagen). Subsequently, 10 fmol of synthetic *Caenorhabditis elegans* miRNA (cel-miR-39-3p; Applied Biosystems) was introduced into the denaturing solution for normalization, following optimized and previously described protocols, prior to RNA extraction [15]. RNA extraction and exosomal miR-1246 quantification was then performed as described in Section 2.5.

### 2.11. Statistical Analysis

Comparisons between experimental groups were performed using Student’s *t*-test. All data are presented as mean ± standard deviation. For the serum samples, the Kolmogorov–Smirnov test was performed to determine normality and significance was subsequently determined using Student’s *t*-test. Statistical analysis was conducted using Prism Software (GraphPad 6.01, La Jolla, CA, USA) and a *p*-value below 0.05 was considered statistically significant.

## 3. Results

### 3.1. MiR-1246 Is Delivered to Recipient Fibroblasts via FN-RMS-Derived Exosomes

**Figure 1:**
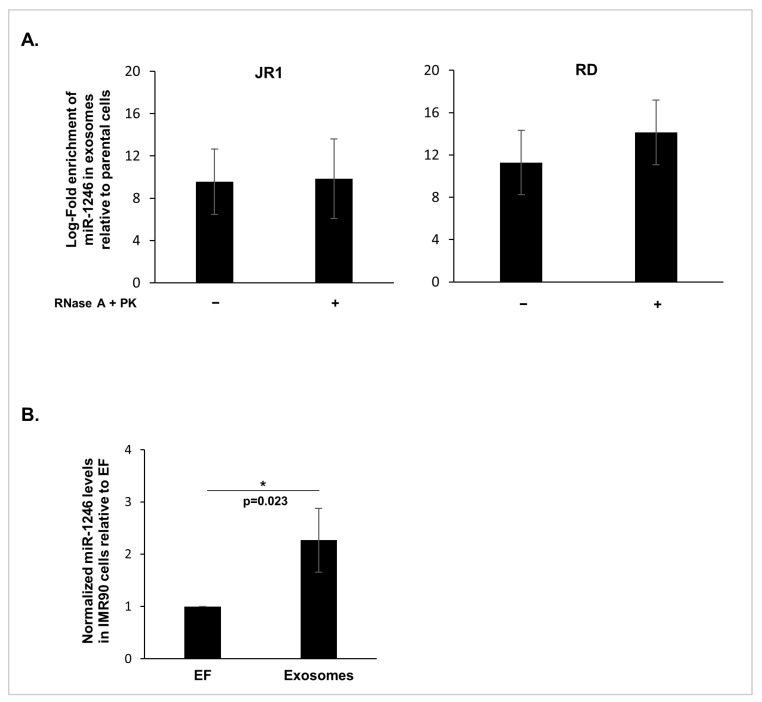
MiR-1246 is encapsulated within FN-RMS-derived exosomes and is delivered to recipient cells. (**A**) Histograms representing the log-fold enrichment of miR-1246 obtained via RTq-PCR in JR1 exosomes ((**left**) panel) and RD exosomes ((**right**) panel) either treated (+) with RNAse A and Proteinase K (PK) or untreated (−), relative to their respective parental cells. Bar graphs represent the mean log-fold enrichment of miR-1246 in exosomes relative to the parental cells ± standard deviation (SD) from 3 independent experiments. (**B**) Histograms representing the quantification of miR-1246 via RTq-PCR in recipient IMR90 fibroblasts following treatment with FN-RMS-derived exosomes relative to IMR90 fibroblasts cultured in exo-free media (EF) used as control. The small RNA RNU6 was used as endogenous control. Bars represent standard deviation and values represent means of at least 3 independent experiments. Asterisk (*) represents significant *p*-value < 0.05 (Student’s *t*-test).

We have previously demonstrated that exosomes derived from both JR1 and RD FN-RMS cells share enrichment in miR-1246 [12]. Since it has been observed that miR-1246 could be found abundantly outside extracellular vesicles, tightly associated with lipoproteins [25], we investigated whether the enriched exosomal miR-1246, as identified by microarray and RTq-PCR [12], was encapsulated within the RMS-derived exosomes. To address this question, exosomes were isolated from basal and untreated JR1 or RD cells, and then, only the exosomes were subjected to two conditions: (i) treatment with RNase and Proteinase K (PK) to eliminate any extravesicular RNA and protein, or (ii) left untreated (Figure 1A). We then performed Taqman RTq-PCR using RNA derived from the two exosomal conditions and their respective parental cells in order to detect the levels of enrichment of miR-1246 in the exosomes. These treatments did not affect miR-1246 levels observed in untreated exosomes and confirmed that miR-1246 was indeed enriched in the RMS-derived exosomes by at least 10-fold, and that miR-1246 was encapsulated inside FN-RMS-derived exosomes as part of their cargo (Figure 1A). Moreover, treating IMR90 cells with 10X JR1-derived exosomes resulted in a significant upregulation in miR-1246 levels (>2-fold) in recipient fibroblasts compared with control IMR90 cells cultured in EF media (Figure 1B). This observation suggests that exosomal miR-1246 is delivered and detected within recipient cells.

### 3.2. Functional Inhibition of miR-1246 in FN-RMS Cells Induces Exosome-Mediated Phenotypical Changes in Recipient Fibroblasts

**Figure 2:**
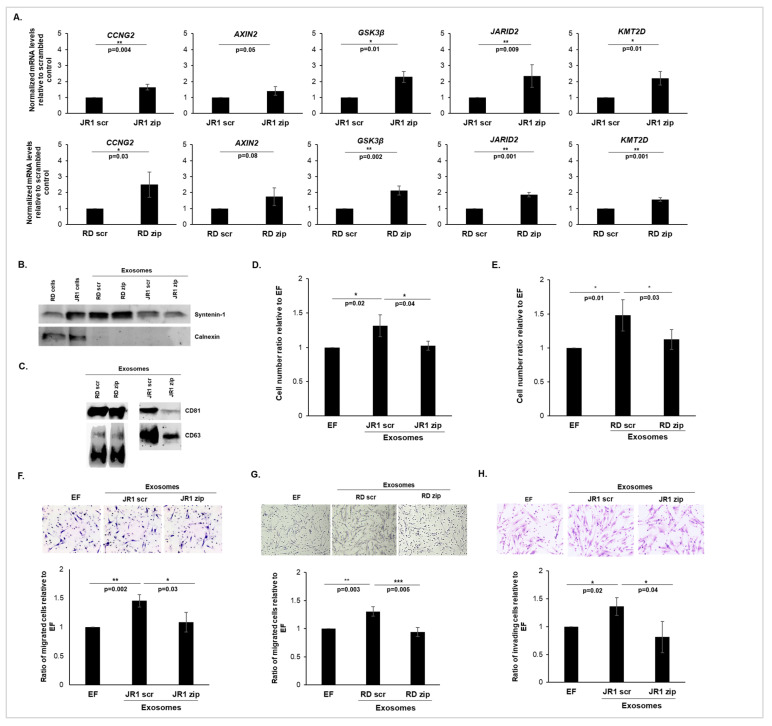
miR-1246 inhibition in FN-RMS cells induces exosome-mediated behavioral changes in recipient fibroblasts. (**A**) Histograms representing the quantification of indicated miR-1246 targets’ expression levels in JR1 zip cells ((**upper**) panel) or RD zip cells ((**lower**) panel) relative to their scrambled controls. L32 was used as an internal control. Representative Western blots for (**B**) syntenin-1 used as a marker of exosomal biogenesis and calnexin used as a negative marker under reducing conditions, and (**C**) CD81 and CD63 used as positive exosomal enriched markers in non-reducing conditions within exosomes derived from transduced JR1 and RD cells. (**D**,**E**) Histograms representing the ratio of viable IMR90 fibroblasts following treatment with exosomes derived from transduced JR1 (**D**) or RD cells (**E**) compared with EF control. (**F**,**G**) Representative images (10× magnification) of H&E-stained fibroblasts and histograms showing the ratio of migrated IMR90 cells treated with exosomes derived from transduced JR1 (**F**) or RD (**G**) cells relative to EF control. (**H**) Representative images (10× magnification) of H&E-stained fibroblasts and histograms showing the ratio of invaded IMR90 cells treated with exosomes from transduced JR1 cells relative to EF control. All bars represent standard deviation from three independent experiments. Asterisks denote *, *p* < 0.05; **, *p* < 0.01; ***, *p* < 0.001 (Student’s *t*-test). The original western blot figures can be found in File S1.

To determine the functional impact of exosomal miR-1246 on recipient cells, we collected exosomes following the functional inhibition of miR-1246 via transducing JR1 and RD cells, using a miRZip lentiviral vector encoding a mature full-length antisense microRNA specifically targeting miR-1246 (JR1-zip and RD-zip). This approach is known to functionally inhibit the target miRNA (miR-1246) without downregulating its expression levels, according to the manufacturer’s instructions. A scrambled vector with an identical backbone and a non-targeting shRNA sequence was used as the control (JR1-scr and RD-scr). The effectiveness of functional miR-1246 inhibition was evaluated through measuring the mRNA expression levels of previously validated miR-1246 targets (Supplementary Table S1), including CCNG2 [26,27,28], AXIN2 [29,30] and GSK3β [29,30], as well as putative miR-1246 targets identified using the TargetScan Database: JARID2 [31] and KMT2D. The results revealed a significant upregulation of all mRNA targets except AXIN2, whose upregulation tended to be significant in JR1-zip (Figure 2A; upper panel) and in RD-zip cells (Figure 2A; lower panel) relative to their respective scrambled controls (JR1-scr and RD-scr), indicating the successful functional inhibition of miR-1246 in our cellular models.

Subsequently, small extracellular vesicles, and in particular exosomes, were isolated from the transduced JR1 and RD cell lines and the efficacy of exosome isolation was confirmed by Western blot analysis (Figure 2B,C), which showed the presence of the well-known specific marker of exosomal biogenesis, syntenin-1 (Figure 2B), as well as CD63 and CD81 (Figure 2C). The negative control calnexin, a cellular protein localized to the endoplasmic reticulum, was, as expected, excluded from the exosomal isolation (Figure 2B). This approach is qualitative in nature and facilitates the identification of exosomal markers within a sample but without providing quantitative information about the amount of these markers present in the sample. Dynamic light scattering further affirmed that the majority of the vesicles exhibited sizes ranging from 53–129 nm in diameter, validating that the isolated particles corresponded to exosomes (Supplementary Figure S1A,B). Quantification of the released exosomes revealed that there was no significant difference in the concentration of exosomes released by JR1-zip or RD-zip cells compared with those released by the scrambled control cells (Supplementary Figure S1C). Treatment of normal IMR90 lung fibroblasts with control JR1-scr- or RD-scr-derived exosomes significantly promoted their proliferation (Figure 2D,E), which is consistent with previous findings showing that RMS-derived exosomes enhanced recipient fibroblast proliferation [12]. Conversely, upon functional inhibition of miR-1246 in JR1 and RD cells and subsequent treatment of fibroblasts with the corresponding exosomes, the effect on proliferation was significantly reversed; thus, the observed increase was abolished (Figure 2D,E). The observed result was also confirmed in WI-38 cells, another human fibroblast cell line (Supplementary Figure S1D), suggesting that exosomal miR-1246 is a key contributor to the exosome-induced proliferation of recipient fibroblasts.

We then assessed the effects of the isolated exosomes on migration and invasion of recipient cells using Transwell assays. We used EF medium in the top chamber to minimize the interference of exogenous factors that might have been present in the complete medium, and serum-free medium in the bottom chamber served to provide a baseline condition for migration and invasion, allowing us to assess the specific effects of exosomes without the confounding influence of serum-derived factors that could have chemoattractant effects [10,12,15]. By maintaining a controlled environment in the upper chamber and utilizing serum-free medium in the lower chamber, we aimed to focus specifically on the influence of exosomes on migration and invasion processes. Thus, treatment of IMR90 fibroblasts with either JR1-scr- or RD-scr-derived exosomes significantly promoted both migration (Figure 2F,G) and the invasion activity (Figure 2H) of recipient cells. This effect was significantly reversed when cells were treated with exosomes from JR1-zip or RD-zip cells, indicating that functional inhibition of miR-1246 in FN-RMS cells inhibits the exosome-mediated promotion of migration and invasion in recipient cells (Figure 2F–H). Together, these results suggest that miR-1246 is an important player within the FN-RMS exosomal cargo, capable of modulating the phenotype of recipient fibroblasts and promoting their aggressiveness.

### 3.3. Delivery of miR-1246 via DOTAP-miRNA Lipoplexes Mimics the Functional Impact of FN-RMS-Derived Exosomes on Recipient Fibroblasts

**Figure 3:**
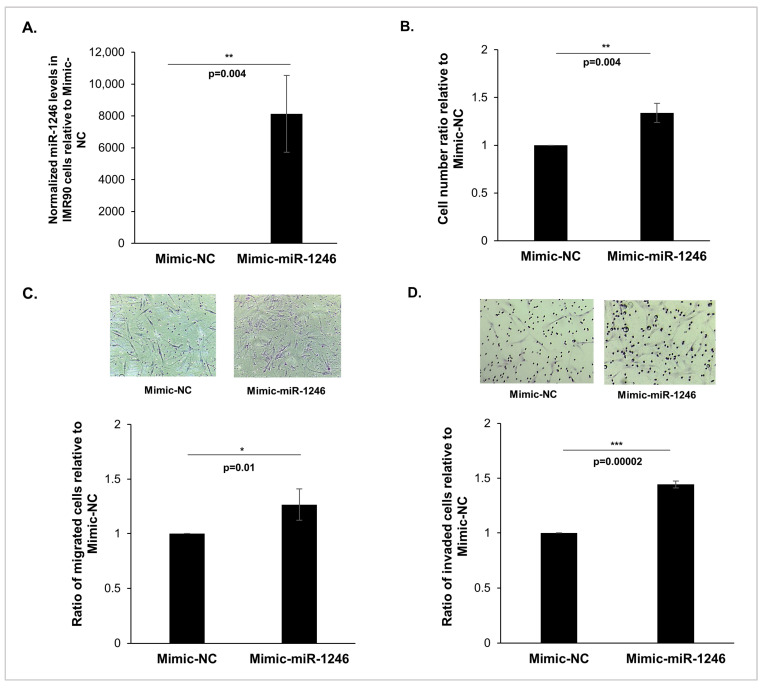
Delivery of miR-1246 induces behavioral changes in recipient fibroblasts. (**A**) Histograms representing the quantification by RTq-PCR of miR-1246 in IMR90 fibroblasts following incubation with DOTAP-miR-1246 mimics (Mimic-miR-1246) relative to DOTAP-negative control (Mimic-NC). Small RNA RNU6 was used as endogenous control. (**B**) Histograms representing the ratio of viable IMR90 fibroblasts following delivery of miR-1246 mimics relative to negative control. (**C**,**D**) Representative images (10× magnification) and histograms showing the ratio of migrated (**C**) and invaded (**D**) IMR90 cells treated with miR-1246 mimics compared to negative control. All bars represent standard deviations from three independent experiments. *, *p* < 0.05; **, *p* < 0.01; ***, *p* < 0.001 (Student’s *t*-test).

Since DOTAP liposomes have been recognized for exhibiting analogous characteristics to exosomes [32], and in order to understand the specific role played by miR-1246 alone in the observed effects of FN-RMS-derived exosomes on recipient fibroblasts, we used DOTAP liposomes to generate DOTAP-miRNA lipoplexes containing miR-1246 mimics. Figure 3A validates that treatment of IMR90 cells with DOTAP-miR-1246 mimics (Mimic-miR-1246) resulted in a significant upregulation in miR-1246 levels compared with cells treated with DOTAP-negative-control lipoplexes (Mimic-NC). Interestingly, delivery of miR-1246-mimic lipoplexes to IMR90 cells significantly enhanced their proliferation (Figure 3B), migration (Figure 3C), and invasion (Figure 3D) relative to IMR90 cells treated with negative control lipoplexes. It is important to note that despite the significant increase in miR-1246 levels in DOTAP-miR-1246-mimic-treated fibroblasts, we observed a slight but significant increase in proliferation, migration, and invasion of these cells compared with negative controls (mimic-NC). This discrepancy suggests that miR-1246 may have complex regulatory effects on cellular processes, and its functional impact may be influenced by various factors including cellular context, target availability, and downstream signaling pathways. Nevertheless, our findings suggest that miR-1246 alone can reproduce the effects of FN-RMS-derived exosomes on recipient cell behavior, underscoring the pivotal role of this miRNA as a key constituent within the FN-RMS exosomal cargo that enhances the aggressiveness of recipient cells.

As a proof of concept to establish that the observed effects were not mediated by free miRNA, we compared the treatment of fibroblasts with DOTAP-mimic lipoplexes to free miR-1246 alone. Treatment of recipient BJ fibroblasts with DOTAP-miR-1246 mimic lipoplexes upregulated the levels of miR-1246 (Supplementary Figure S2A, left panel) and significantly induced the migration of BJ fibroblasts compared with DOTAP-control-mimic lipoplexes (Supplementary Figure S2B, left panel). On the other hand, treatment with free miR-1246 mimics alone did not deliver miR-1246 to recipient fibroblasts, resulting in no discernible effect on the migration of these cells (Supplementary Figure S2A,B, right panels). This demonstrates that vesicle-mediated carrying of miR-1246 is mandatory for its successful delivery to recipient cells and its subsequent functional impact on recipient fibroblasts.

### 3.4. MiR-1246 Modulates Important Downstream Targets in Recipient Fibroblasts

**Figure 4:**
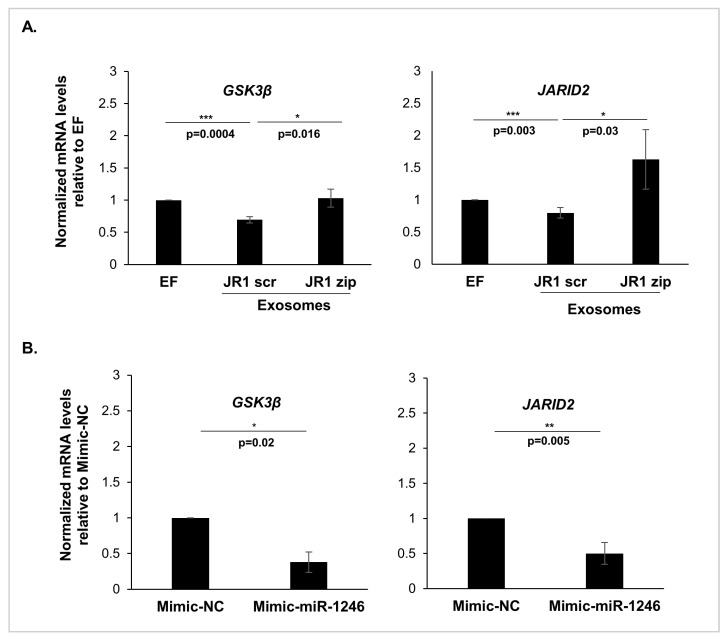
Delivery of miR-1246 regulates downstream targets in recipient IMR90 fibroblasts. (**A**) Quantification by qRT-PCR of miR-1246 targets’ mRNA levels in IMR90 cells treated with exosomes from transduced JR1 cells relative to control (EF). (**B**) Quantification by qRT-PCR of miR-1246 targets’ mRNA levels in IMR90 cells treated with Mimic-miR-1246 lipoplexes relative to control cells treated with negative control mimic lipoplexes (Mimic-NC). L32 was used as endogenous internal control. Bars represent standard deviations while asterisks denote a significant *p*-value from 3 independent experiments. *, *p* < 0.05, **, *p* < 0.01, ***, *p* < 0.001 (Student’s *t*-test).

To investigate potential downstream signaling pathways that may elucidate the observed changes in recipient fibroblast behavior, we evaluated the expression levels of miR-1246 targets in IMR90 cells treated with either exosomes (Figure 4A) or DOTAP-miR-1246 mimics (Figure 4B). A significant downregulation in *GSK3β* and *JARID2* mRNA levels was validated following treatment with JR1-scrambled derived exosomes (Figure 4A). This effect was reversed following the functional inhibition of miR-1246 in JR1 cells and treatment with the resulting exosomes (Figure 4A). Similarly, exposure of IMR90 cells to DOTAP-miR-1246 mimics resulted in a significant downregulation of *GSK3β* and *JARID2* compared with those treated with DOTAP-negative control mimics (Figure 4B).

GSK3β is a critical regulator within the Wnt signaling pathway, acting through the phosphorylation of β-catenin as a key step for its rapid proteasomal degradation [33]. Upon activation of the Wnt pathway, GSK3β is inhibited, allowing the accumulation of β-catenin, its translocation to the nucleus, and subsequent binding to DNA [34,35]. As the Wnt pathway has been associated with tumorigenesis [36], we aimed to explore whether exosomal miR-1246 could contribute to the activation of β-catenin-dependent pathways.

**Figure 5:**
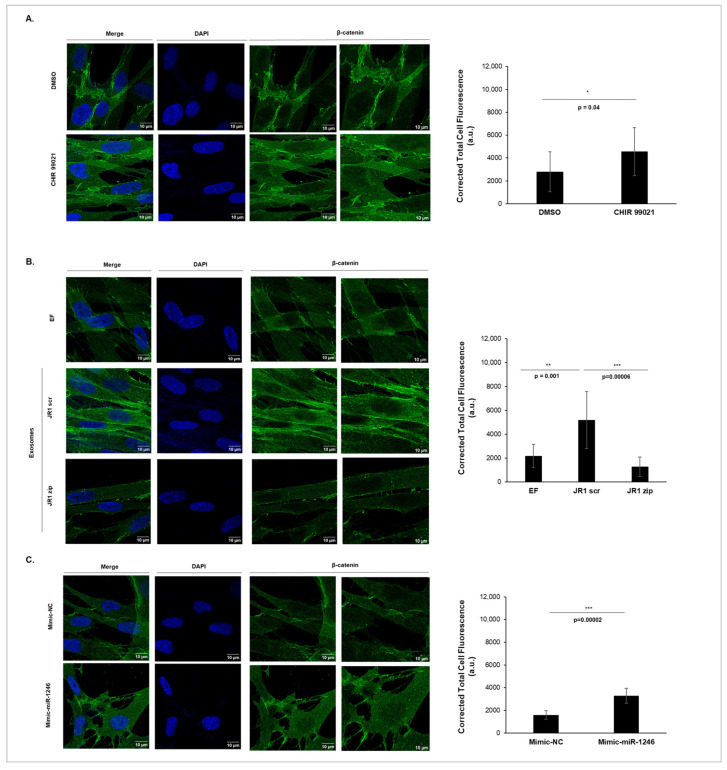
Delivery of miR-1246 induced β-catenin cellular expression and its nuclear accumulation. (**A**–**C**) Immunofluorescence microscope images (40× magnification; final column represents zoomed-in images with zoom set at 100% on ImageJ) showing the localization of β-catenin (green) and histograms of the quantification of the corrected total cell fluorescence (CTCF) of β-catenin inside the nucleus in IMR90 cells, either (**A**) treated with potent GSK3β inhibitor (CHIR99021, 3 µM) as a positive control, DMSO was used as control for CHIR99021, or (**B**) EF media treated with exosomes derived from transduced JR1 cells, or (**C**) treated with DOTAP-mimic lipoplexes. DAPI was used to stain cell nuclei (blue). Bars represent standard deviations of CTCFs from 3 independent experiments. *, *p* < 0.05; **, *p* < 0.01; ***, *p* < 0.001 (Student’s *t*-test).

Immunofluorescence analysis was used to detect the subcellular localization of β-catenin. CHIR99021, a potent GSK3β inhibitor known to activate the Wnt pathway [37], was used as a positive control to validate our experiment, while DMSO served as the negative control (Figure 5A). As expected, treatment of IMR90 cells with CHIR99021 (3 µM) led to an overall increase in the expression levels of β-catenin, with a significant accumulation in the nucleus compared with the control DMSO (Figure 5A). Figure 5B shows that β-catenin was mainly present in the cytoplasm of untreated IMR90 cells (EF). Upon treatment with JR1-scrambled exosomes (JR1 scr), we observed an overall increase in the expression levels of β-catenin in the cell, with a significant accumulation in the nucleus compared with the control (EF), as evidenced by the corrected total cell fluorescence signal of nuclear β-catenin (Figure 5B). This suggests a possible deregulation of the Wnt pathway upon exposure to these exosomes. In contrast, exosomes isolated from JR1-miRZip transduced cells (JR1 zip) did not induce the cellular accumulation of β-catenin or its translocation to the nucleus of the treated cells (Figure 5B). Moreover, we observed increased expression levels of β-catenin within the cell, accompanied by its significant nuclear accumulation upon delivery of miR-1246 mimics (Mimic-miR-1246) compared with DOTAP-control mimics (Figure 5C). Altogether, both JR1 exosomes containing functional miR-1246 and the direct delivery of miR-1246 via miRNA-mimic lipoplexes promoted the nuclear translocation of β-catenin, implying a potential involvement of miR-1246 in Wnt pathway deregulation. These results suggest that exosomal miR-1246 promotes phenotypic changes in recipient IMR90 fibroblasts that could be mediated by a deregulation in the Wnt signaling pathway.

### 3.5. High miR-1246 Levels Are Detected in Serum-Derived Exosomes of RMS Patients

**Figure 6:**
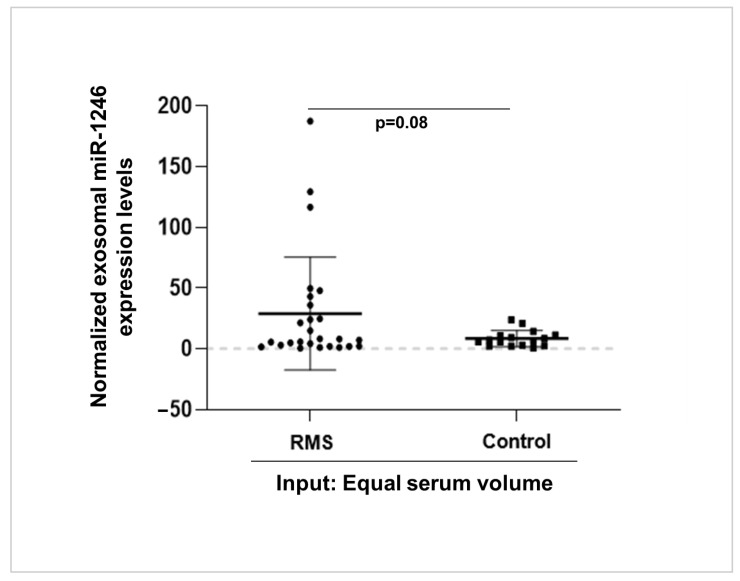
Exosomal miR-1246 expression in human RMS patient serum samples. The whisker plot represents the quantification of miR-1246 levels in exosomes isolated from serum of RMS patients (*n* = 26) and controls (*n* = 16) by qRT-PCR. Equal serum volume was used as input and *C. elegans* miR-39-3p (Cel-miR-39-3p) spike-in control was used for normalization; *p*-value was calculated using Student’s *t*-test.

To assess the clinical relevance of our in vitro findings, we performed a pilot clinical investigation aiming at assessing the presence of exosomal miR-1246 in the sera of RMS patients. To achieve this, we isolated exosomes from the serum of 26 RMS patients as well as from 16 age-matched controls. The results exhibit a trend towards significance (*p* = 0.08), indicating potentially higher levels of miR-1246 in serum exosomes isolated from RMS patients compared with those from controls (Figure 6). Due to the small number of sera samples, no specific clinical features could be associated with the samples with increased expression in this cohort. While this finding requires validation in a larger and more comprehensive cohort, it implies a potential association between RMS and elevated levels of exosomal miR-1246 in the serum.

## 4. Discussion

The aggressive nature of RMS poses a significant challenge to the success of treatments, prompting the exploration of novel non-invasive therapeutic strategies. Given that FN-RMS constitutes 80% of all RMS cases, the deciphering of molecular events sustaining aggressiveness and identification of markers capable of distinguishing between various prognostic and survival outcomes within this substantial subgroup is crucial [38]. Exosomes are emerging as promising candidates in both cancer treatment and monitoring due to their easy isolation using non-invasive techniques, reduced toxicity, and efficient drug-delivery capabilities [39]. In a previous study, we demonstrated that FN-ERMS-derived exosomes enhanced the invasive properties of recipient cells, including both fibroblasts and endothelial cells, thereby reshaping recipient cell behavior to favor tumor progression [12]. Moreover, RMS-derived exosomes were found to carry enriched levels of miRNA compared with RMS cells [12]. These exosomal miRNAs could potentially play a role in exosome-mediated effects, suggesting their potential use in treatment or non-invasive diagnostic strategies. Notably, miR-1246 was consistently enriched in all RMS-derived exosomes [12], indicating its relevance for further investigation. MiR-1246 has been shown to contribute to cancer growth, invasion, and metastasis, mostly acting as an oncomiR with biological significance in the pathoetiology of different types of cancer [20]. Current studies mostly demonstrate that its levels of expression correlate with advanced cancer stages or resistance to treatment [21,22,23], but there has been little investigation into the specific effects of exosomal miR-1246 on the cells of the tumor microenvironment. MiR-1246 has never been investigated in RMS in general nor in FN-RMS in particular. Our work shows that miR-1246 is, in fact, encapsulated inside FN-RMS-derived exosomes, pinpointing its putative key role in RMS progression. Our finding is supported by the work of Xu et al., who demonstrated that certain miRNA species are selectively and highly enriched in pancreatic cancer exosomes, with miR-1246 being the most abundant [40]. Furthermore, miR-1246 was identified in salivary exosomes as a potential biomarker for pancreatobiliary tract cancer, highlighting its diagnostic potential [41]. Altogether, those studies and our work collectively support the notion that miR-1246 encapsulated within exosomes plays a crucial role in cancer progression and intercellular communication.

Our findings provide valuable insights into the functional impact of exosomal miR-1246 derived from FN-RMS cells on recipient cells. We have demonstrated that functional inhibition of miR-1246 in FN-RMS cells and treatment of normal fibroblasts with their derived exosomes reversed the exosome-induced proliferation, migration, and invasion of recipient cells, suggesting that exosomal miR-1246 contributes to modulating the phenotype of recipient fibroblasts and promoting their aggressiveness. Since inhibiting a miRNA may lead to myriad changes inside cells due to modulating multiple downstream targets that may subsequently be modified within the exosomal cargo [42,43], we demonstrated that the delivery of miR-1246 alone via DOTAP-mimic lipoplexes (but not free miR-1246) is capable of promoting similar phenotypic changes. This observation suggests a potential direct impact of miR-1246 delivery to recipient cells, indicating a specific role in FN-RMS-derived exosome-mediated effects on normal cells. Studies have demonstrated that exosomal miR-1246 plays a role in promoting cell proliferation, invasion, and drug resistance in breast cancer and mutant p53 cancers [27,44]. Thus, our work supports the notion that even in a rare cancer such as FN-RMS, the enrichment of miR-1246 in exosomes may not be a random process but rather a cancer-cell mechanism to promote cancer progression.

Activation of Wnt signaling and subsequent β-catenin transcriptional activity has been shown to promote cell proliferation, migration, and invasion, which can enhance cancer progression [45,46,47]. Studies have demonstrated that miR-1246 activates the Wnt/β-catenin signaling pathway by directly targeting *GSK3β* [21,29]. Consistently, our results show that treatment with FN-RMS-derived exosomes or with miR-1246-mimic lipoplexes downregulates *GSK3β* in recipient fibroblasts while upregulating nuclear accumulation of β-catenin. Importantly, functional inhibition of miR-1246 in FN-RMS cells reverses these effects, suggesting the importance of Wnt signaling in recipient cells as a mechanism of action for this miRNA in promoting the observed changes. Altogether, our data suggest that exosomal-miR1246-driven molecular mechanisms may involve activation of the Wnt pathway. However, this mechanism is probably not exclusive and the observation that FN-RMS-derived exosomes can decrease *JARID2* expression also paves the way for a possible impact of miR-1246 on the epigenome. JARID2 interacts with the polycomb repressive complex 2 (PRC2) thus impacting H3K27me3 and the cell’s transcriptome [48]. JARID2 has been implicated in tumorigenesis [49,50]. While JARID2 may have a tumor-suppressive role in FP-RMS [51], its impact on the epigenome of recipient cells and subsequent functional changes in RMS is unclear. Future studies aiming at investigating miR-1246’s effect on the epigenome in recipient fibroblasts by downregulating players like *JARID2* are, thus, of utmost interest.

Access to clinical samples from RMS patients poses a significant challenge due to the rarity of this disease. Investigation of exosomal miRNA in RMS patients has, to our knowledge, been investigated in only one study with a limited number of RMS patients’ sera (n = 6) [15]. Our pilot clinical investigation revealed a trend towards significantly higher levels of miR-1246 in serum exosomes isolated from RMS patients compared with those from control patients. This finding suggests a potential association between RMS and elevated levels of exosomal miR-1246 in the serum, supporting a putative functional role of exosomal miR-1246, in accordance with our in vitro data. The size of our current cohort did not allow it to be stated whether any correlation exists between the presence of exosomal miR-1246 in RMS patients’ serum and markers of disease aggressiveness, and future work is thus needed to investigate this question.

Several studies have demonstrated the potential of exosomal miRNAs in general and exosomal miR-1246 in particular as biomarkers for early diagnosis, disease aggressiveness, and subtype classification in gastric cancer, breast cancer, colon cancer, and esophageal squamous cell carcinoma [52,53,54,55,56]. Additionally, using exosomal miRNAs as biomarkers are potentially less invasive options in disease monitoring since exosomes can be isolated in high quality from small quantities of biological fluids, including patient sera, using nanotechnology and microfluidic devices [9,55,57]. These findings suggest that exosomal miRNAs hold promise as non-invasive biomarkers for cancer detection and characterization. Moreover, the diagnostic potential of a combined panel of protein and miRNA serum–exosome biomarkers was demonstrated for pancreatic cancer [58], and may therefore be useful in other tumors as well. Additionally, the study by Chen et al. (2021) highlighted the diagnostic value of serum miR-1246 in hepatocellular carcinoma, further supporting the potential of exosomal miR-1246 as a biomarker for cancer diagnosis [59]. These studies collectively underscore the significance of exosomal miR-1246 as a potential biomarker for cancer diagnosis, and a driver of oncogenic properties. Further validation and quantification of exosomal miR-1246 in larger patient cohorts will be important to assess its potential usefulness in this setting.

## 5. Conclusions

In conclusion, our study sheds light on the potential role of miR-1246-enriched exosomes in promoting paracrine signaling in FN-RMS, with a functional impact on promoting oncogenic properties in recipient cells. We demonstrate that functional inhibition of miR-1246 in FN-RMS cells reverses the exosome-mediated effects on recipient fibroblasts, suggesting a central role in modulating the phenotype of these cells and promoting their aggressiveness. The delivery of miR-1246-mimic lipoplexes replicates the phenotypic changes induced by FN-RMS-derived exosomes, indicating a potential direct impact of miR-1246 on recipient cells. Mechanistically, we found that miR-1246 impacts the Wnt/β-catenin signaling pathway in exosome-treated fibroblasts, but this does not ascertain whether this signaling pathway is the main contributor to the observed phenotypic effects. A pilot cohort of sera from patients with RMS showed a trend towards higher exosomal miR-1246 levels in serum when compared with a separate control cohort, suggesting its origin from RMS tumors in these patients. In summary, our study provides insights into a role for exosomal miR-1246 in paracrine signaling by FN-RMS. Further investigations will be needed to identify targetable downstream signaling effectors, and to validate the role of these observed phenotypic changes in recipient cells on clinical tumor growth and invasion using in vivo preclinical models of RMS invasion and metastasis.

## Data Availability

The data and materials used in this study are available once published and upon request.

## Supplementary Materials

The following supporting information can be downloaded at: https://www.mdpi.com/article/10.3390/cancers16091652/s1, Table S1: List of previously identified miR-1246 targets and their validation. Figure S1: miR-1246 inhibition reduces exosome-mediated proliferation of recipient WI-38 fibroblasts; Figure S2: Liposome-mediated delivery of miR-1246 is necessary for its functional effects; File S1: The original western blot figures. References [19,22,26,27,28,29,30,31] are cited in the Supplementary Materials.
