# Supplementary material for: Exosome-Mediated Paracrine Signaling Unveils miR-1246 as a Driver of Aggressiveness in Fusion-Negative Rhabdomyosarcoma
Source: Cancers (Basel). 2024 Apr 25;16(9):1652. doi: 10.3390/cancers16091652 (PMC11083369; doi:10.3390/cancers16091652)
Supplement: Supplementary file 1 [file cancers-16-01652-s001.zip › cancers-2898162-supplementary.pdf]

Supplementary Materials

Supplementary Table S1. List of previously identified miR-1246 targets and their validation.

**MiR-1246 target**	**Validation technique**	**Reference**
CCNG2	**Luciferase reporter assay, RTq-PCR, Western Blot**	**[19, 26-28]**
AXIN2	**Luciferase reporter assay, RTq-PCR, Western Blot**	**[29-31]**
GSK3β	**Luciferase reporter assay, RTq-PCR, Western Blot**	**[29-31]**
JARID2	**RTq-PCR**	**[22,31]**
KMT2D	**Putative (predicted) target**	**TargetScan**

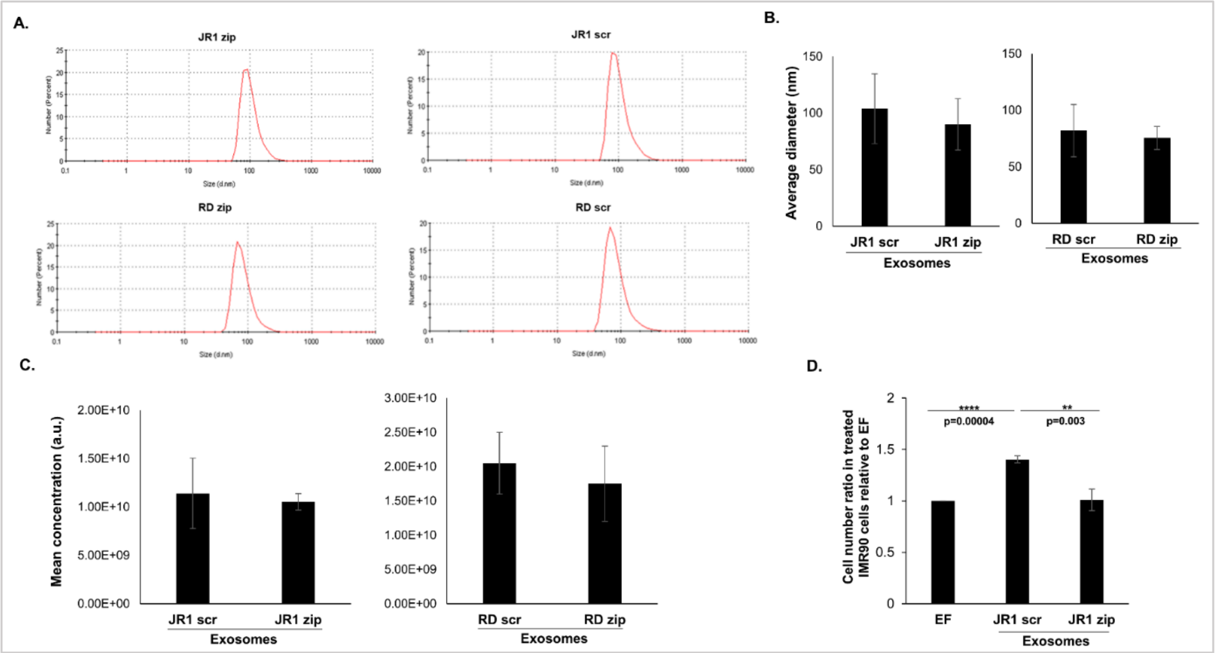

Supplementary Figure S1. miR-1246 inhibition reduces exosome-mediated proliferation of recipient WI-38 fibroblasts. (A) size distribution by number curves of exosomes derived from transduced JR1 cells (upper panel) or RD cells (lower panel) obtained by dynamic light scattering. A representative of each sample is indicated. Graphs are representatives of at least 3 independent exosome isolations and size quantification. (B) Bar graphs of the calculated average diameter of the isolated JR1 and RD exosomes from at least 3 independent exosome isolations. Bars represent standard deviation. (C) Bar charts representing the mean concentration (a.u.) of the isolated JR1 ord RD exosomes from 3 independent exosome isolations. Bars represent standard deviation from the mean. (D) Bar charts representing the ratio of viable IMR90 fibroblasts following treatment with exosomes derived from the different transduced JR1 cells relative to control (EF). Values represent ratio of means of at least 3 independent experiments. Bars represent standard deviation. Asterisks ** represent $p < 0.01$, **** represent $p < 0.001$ compared to control condition (Student t-test).

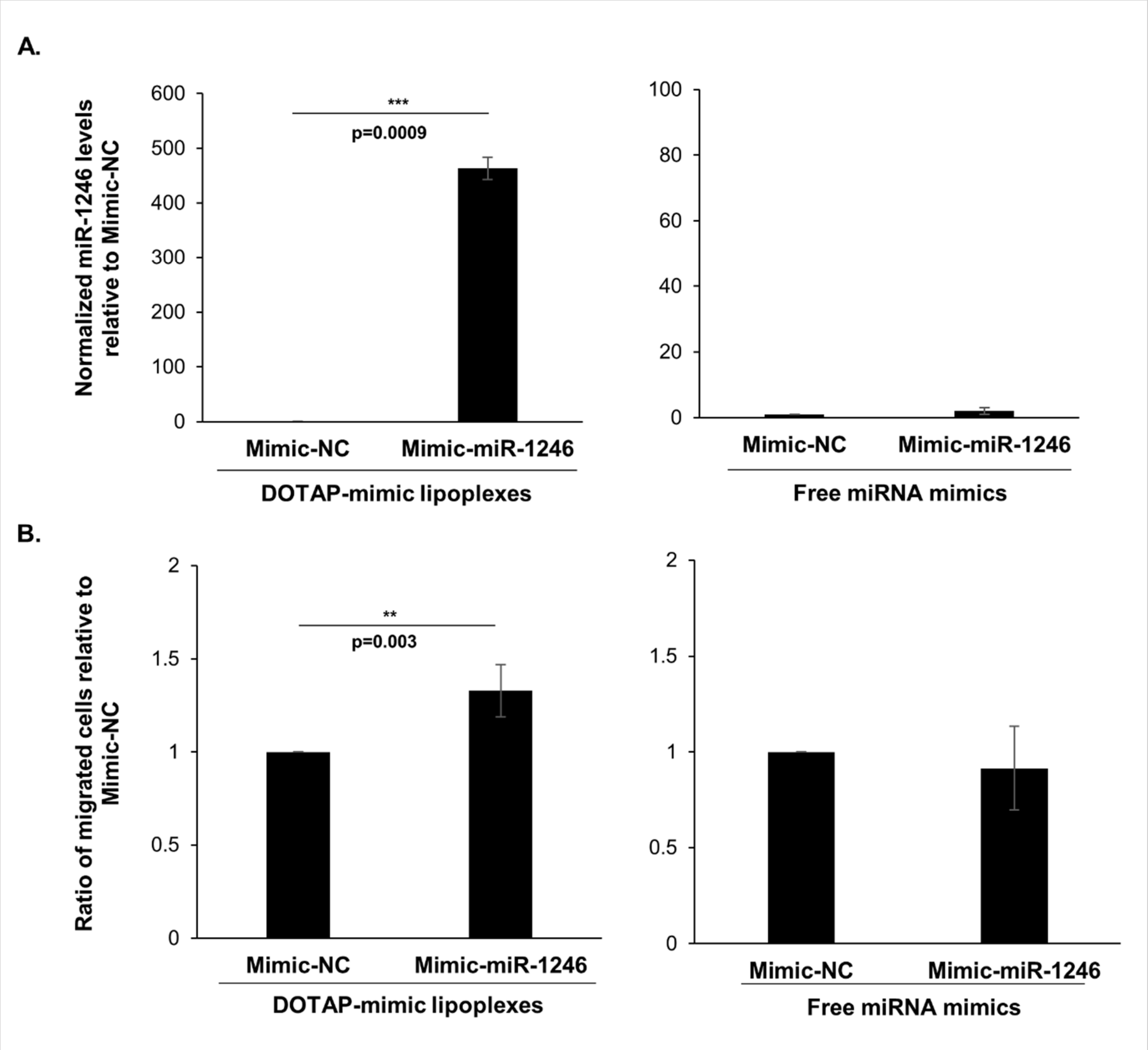

Supplementary Figure S2. Liposome-mediated delivery of miR-1246 is necessary for its functional effects. (A) Quantification by RTq-PCR of miR-1246 in BJ fibroblasts following transfection with miR-1246 mimics (Mimic-miR-1246) relative to negative control mimics (Mimic-NC) (left panel) or by free miR-1246 mimics compared to free control mimics (right panel). The small RNA RNU6 was used as endogenous control. (B) Bar graph showing the ratio of migrated BJ cells following treatment with DOTAP-miR-1246 mimics (left panel) or free miR-1246 mimics (right panel) relative to DOTAP-control or free control mimics, respectively. Bars represent standard deviations from 3 independent experiments. Asterisks ** denote $p < 0.01$, *** $p < 0.001$ (Student t-test).
