# Supplementary material for: Exosome-Mediated Paracrine Signaling Unveils miR-1246 as a Driver of Aggressiveness in Fusion-Negative Rhabdomyosarcoma
Source: Cancers (Basel). 2024 Apr 25;16(9):1652. doi: 10.3390/cancers16091652 (PMC11083369; doi:10.3390/cancers16091652)

CD81 – Positive marker for exosomes

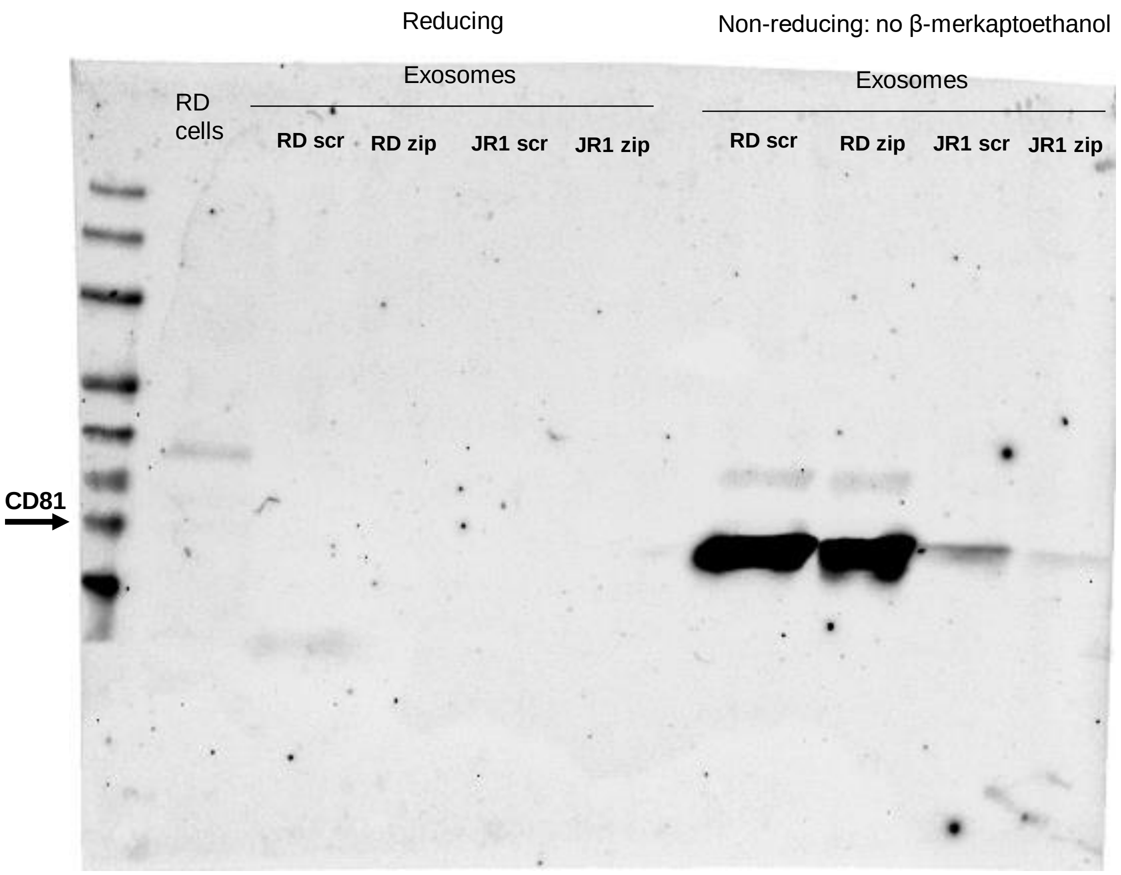

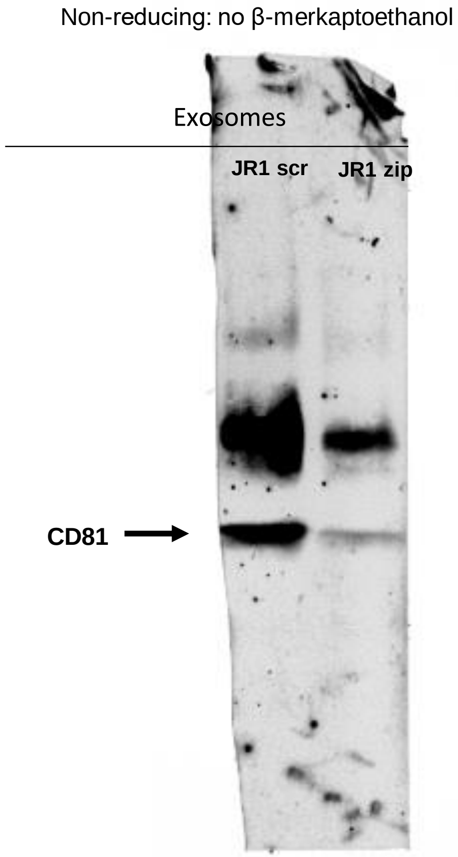

The membrane was cut to view JR1 scr and JR1 zip exosomes at higher exposure

CD63 – Positive marker for exosomes

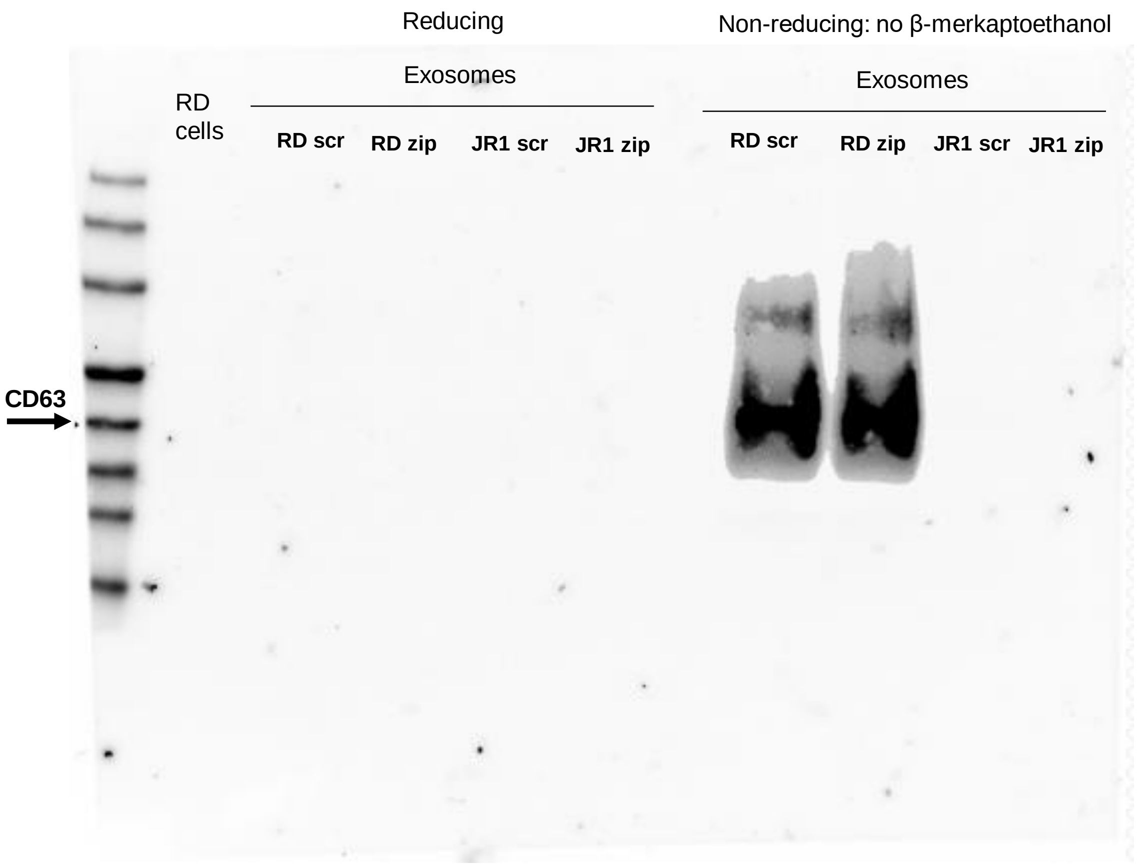

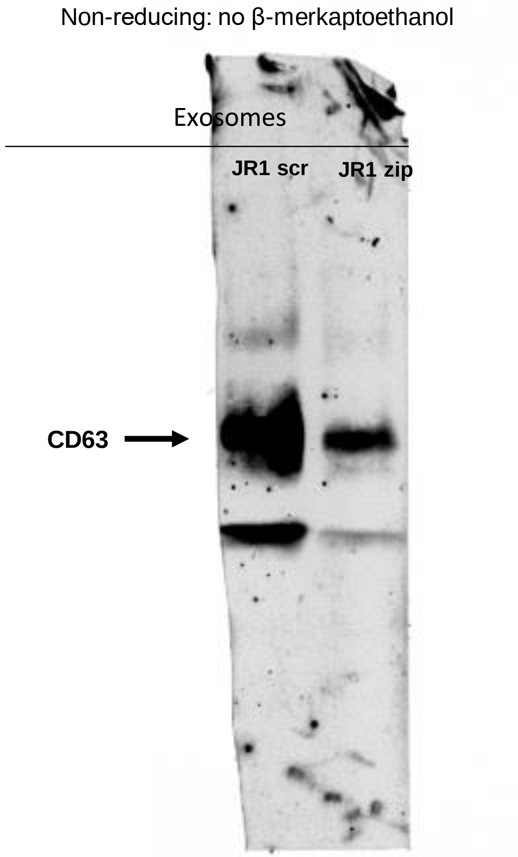

The membrane was cut to view JR1 scr and JR1 zip exosomes at higher exposure

Syntenin-1 – Positive marker for exosomes

Reducing conditions

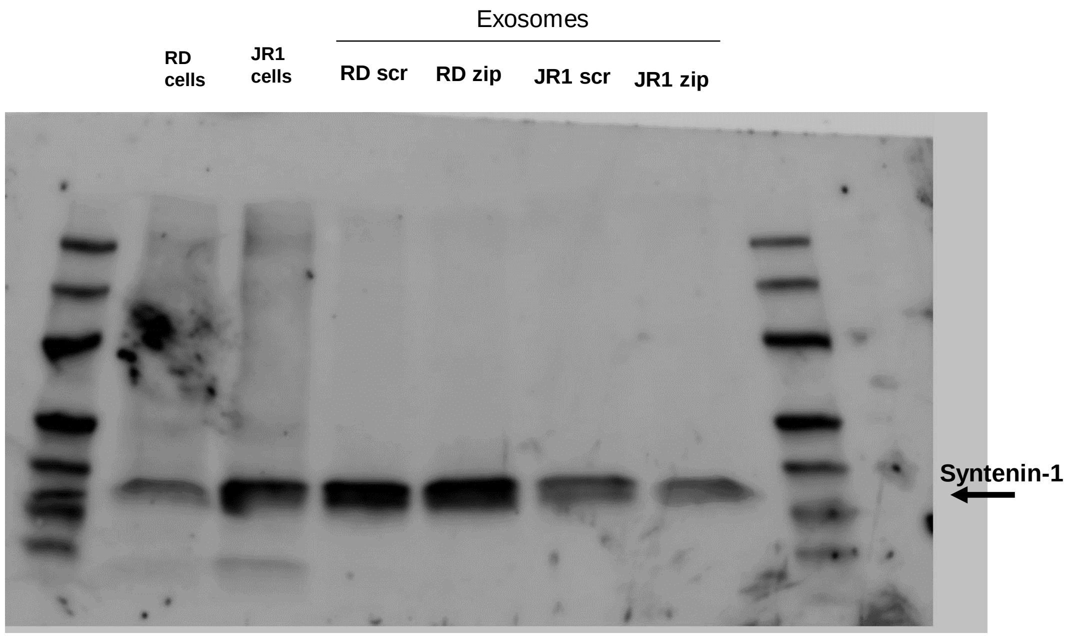

Calnexin – Negative marker for exosomes

Reducing conditions

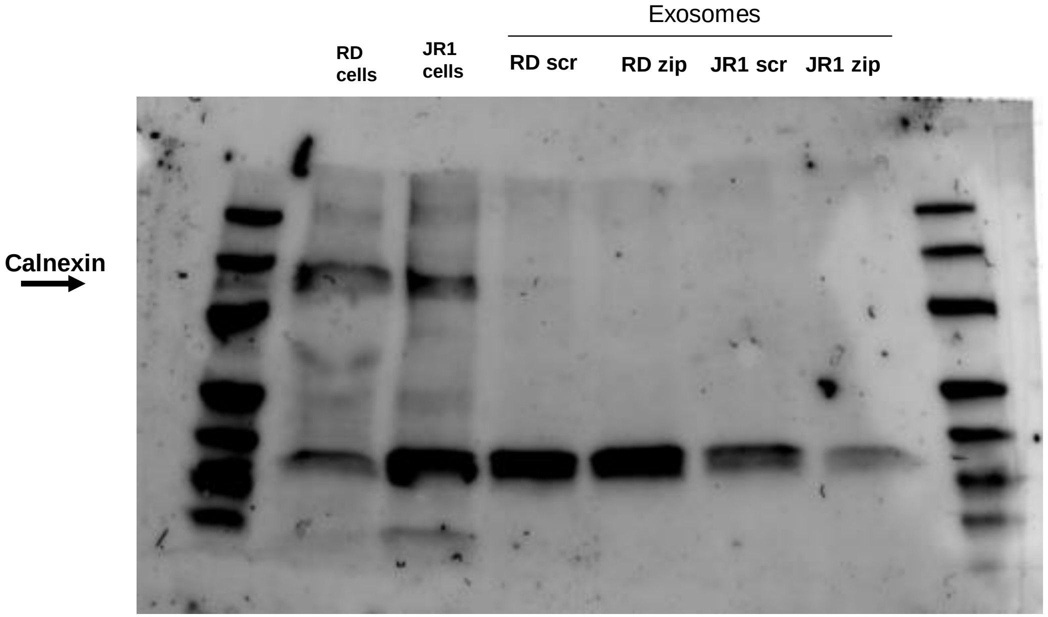

Supplement: Supplementary file 1 [file cancers-16-01652-s001.zip › File S1. The original western blot figures .pdf]
